# C-di-GMP Hydrolysis by *Pseudomonas aeruginosa* HD-GYP Phosphodiesterases: Analysis of the Reaction Mechanism and Novel Roles for pGpG

**DOI:** 10.1371/journal.pone.0074920

**Published:** 2013-09-16

**Authors:** Valentina Stelitano, Giorgio Giardina, Alessandro Paiardini, Nicoletta Castiglione, Francesca Cutruzzolà, Serena Rinaldo

**Affiliations:** 1 Istituto Pasteur-Fondazione Cenci Bolognetti, Department of Biochemical Sciences, Sapienza University of Rome, Rome, Italy; 2 Department of Biochemical Sciences, Sapienza University of Rome, Rome, Italy; The Scripps Research Institute and Sorrento Therapeutics, Inc., United States of America

## Abstract

In biofilms, the bacterial community optimizes the strategies to sense the environment and to communicate from cell to cell. A key player in the development of a bacterial biofilm is the second messenger c-di-GMP, whose intracellular levels are modulated by the opposite activity of diguanylate cyclases and phosphodiesterases. Given the huge impact of bacterial biofilms on human health, understanding the molecular details of c-di-GMP metabolism represents a critical step in the development of novel therapeutic approaches against biofilms. In this study, we present a detailed biochemical characterization of two c-di-GMP phosphodiesterases of the HD-GYP subtype from the human pathogen *Pseudomonas aeruginosa*, namely PA4781 and PA4108. Upstream of the catalytic HD-GYP domain, PA4781 contains a REC domain typical of two-component systems, while PA4108 contains an uncharacterized domain of unknown function. Our findings shed light on the activity and catalytic mechanism of these phosphodiesterases. We show that both enzymes hydrolyse c-di-GMP in a two-step reaction *via* the linear intermediate pGpG and that they produce GMP *in vitro* at a surprisingly low rate. In addition, our data indicate that the non-phosphorylated REC domain of PA4781 prevents accessibility of c-di-GMP to the active site. Both PA4108 and phosphorylated PA4781 are also capable to use pGpG as an alternative substrate and to hydrolyse it into GMP; the affinity of PA4781 for pGpG is one order of magnitude higher than that for c-di-GMP. These results suggest that these enzymes may not work (primarily) as genuine phosphodiesterases. Moreover, the unexpected affinity of PA4781 for pGpG may indicate that pGpG could also act as a signal molecule in its own right, thus further widening the c-di-GMP-related signalling scenario.

## Introduction

C-di-GMP (3'–5'-diguanylic acid) is a ubiquitous intracellular second messenger, which is crucial for the physiology and pathogenesis of a variety of bacteria, including those of clinical relevance. C-di-GMP regulates complex prokaryotic processes such as virulence, motility and biofilm formation [1,2,3,4,5]. The possibility to defeat bacteria growing in biofilms by targeting c-di-GMP signalling is a challenging issue, given the huge impact of biofilms on human health [6,7].

The switch from the planktonic to the sessile way of life (typical of biofilms) is achieved when, in response to environmental stimuli, the intracellular levels of c-di-GMP reach a threshold thus inducing the expression of key genes, as well as the formation of extracellular polymeric substance [6,8,9].

The intracellular levels of c-di-GMP are modulated by the opposite activity of diguanylate cyclases (DGCs), which synthesize c-di-GMP (from two GTP molecules), and phosphodiesterases (PDEs), which hydrolyse c-di-GMP producing linear 5’ phosphoguanylyl-(3’–5’)-guanosine (pGpG) or GMP. DGCs are also called GGDEF proteins after their conserved active site motif; on the other hand, PDEs contain EAL or HD-GYP domains [10,11,12]. The HD-GYP domain contains a HHExxDGxxGYP motif and is unrelated to the EAL domain [13]. The significance of having two types of unrelated c-di-GMP hydrolases in the bacterial genomes is still matter of debate [14], also considering that HD-GYP PDEs are not ubiquitous.

The presence of a variety of genes coding for GGDEF, EAL, HD-GYP or GGDEF-EAL proteins in the genome of many bacterial species [11] indicates that bacteria regulate c-di-GMP turnover and biofilm formation in an extremely sophisticated manner. Most of these enzymes are indeed associated with known or hypothetical signal input domains, which are putatively involved in sensing a range of environmental signals (oxygen, blue light, nutrient starvation, antibiotics, etc.) [15]. However, how DGCs and PDEs function together to produce a coherent output signal is still unclear; different c-di-GMP circuits could be separate in time and in space, through compartmentalization [16,17,18].

To date, few biochemical data on the proteins involved in c-di-GMP turnover are available, particularly on HD-GYP PDEs [19,20,21,22]. As mentioned above, the HD-GYP domain is widespread but not ubiquitous in bacteria (over 1000 genes have been found among the whole sequenced bacteria genomes [7]). This domain is classified as a metal-dependent phosphohydrolase; a divalent cation (most likely Mg^2+^ or Mn^2+^) is required for catalysis [23,24] but the molecular mechanism of action is still unknown.

Among PDEs, several structures of the EAL subtype are available [25,26,27], contrary to the HD-GYP counterpart, where only one structure is available [28]. Lovering and co-workers recently solved the structure of Bd1817, an unconventional HD-GYP protein from the predatory bacterium *Bdellovibrio* *bacteriovorus* , which was found to be inactive as PDE [28]. The fold and active site of the HD-GYP domain are different from those of EAL proteins, and restricted access to the active-site cleft is indicative of a different mode of regulation of the activity. The region encompassing the GYP motif has a novel conformation, is surface exposed and is available for complexation with binding partners, including GGDEF proteins [28], as seen for RpfG [29].

RpfG from *Xanthomonas campestris* (XC_2335) represents the archetype of this class of enzymes, since its characterization demonstrated for the first time the specific involvement of the HD-GYP domain in c-di-GMP degradation [13]; in this study, Ryan and coworkers show that expression of RpfG fully complements the mutation of EAL-type PDE in *X. campestris* and that the purified protein has c-di-GMP-specific PDE activity, whose product is GMP. Mutation of the *rpfG* gene leads to a decrease in the synthesis of virulence factors (including extracellular enzymes), alteration in biofilm formation, decreased pilus dependent motility and virulence [30].

In addition to *Xanthomonas* RpfG, genetic and (preliminary) molecular data are available on representatives from *Pseudomonas* and *Borrelia* [19,22]. In *Borrelia burgdorferi*, PdeB, the sole HD-GYP PDE found in this genome, controls motility and virulence; mutation of this gene hampers the transmission of infection to mammalian hosts, thus confirming that also low c-di-GMP levels play a key role in the infection process [22].

In *P. aeruginosa*, 3 genes harbour HD-GYP domains: PA4108, PA4781 and PA2572. The first two proteins control the swarming motility and the production of virulence factors and were shown to have PDE activity *in vivo*, since their mutation leads to increased levels of c-di-GMP [19]. Moreover, the corresponding HD-GYP domains were able to complement the mutation of the RpfG gene (encoding a HD-GYP PDE) in *X. campestris* [19]. On the other hand, the role of the third protein PA2572 (which has a different YN-GYP signature) is uncertain [19]: this protein is inactive in c-di-GMP hydrolysis and it has a cryptic negative influence on swarming. Nevertheless, all three proteins regulate virulence of *P. aeruginosa* since their mutation leads to a reduction of bacterial virulence in the larvae of *Galleria* *mellonella* [19]. Due to their importance in virulence and since the biochemical and structural properties of HD-GYPs are still poorly understood, a deep characterization of this class of proteins is highly desirable.

In this study, we present a detailed characterization of the two active PDEs from *P. aeruginosa*, namely PA4108 and PA4781, following two parallel strategies. On one hand, we carried out complementation assays in *E. coli*, a bacterium known to lack HD-GYP-containing proteins, in order to assess whether the *P. aeruginosa* PDEs are active in a HD-GYP-free background. On the other hand, we have studied the enzymatic activity *in vitro*, thus clarifying that pGpG is an intermediate in the hydrolysis of c-di-GMP carried out by HD-GYP phosphodiesterases. The kinetic and bioinformatic data also indicate that PA4781 binds pGpG with higher affinity with respect to c-di-GMP, thus suggesting a novel and intriguing role for this linear dinucleotide.

## Methods

### Cloning and site-direct mutagenesis

Synthetic PA4781 and PA4108 genes were purchased from Geneart, subcloned into the pET28b and pET24 vectors in frame with a N-terminal and C-terminal His-tag, respectively (GENEART). A truncated version of PA4781 (hereinafter PA4781_HD-GYP_) was produced by PCR, starting from PA4781 as a template, in order to remove the first 111 residues, corresponding to the REC domain. The corresponding fragment was then cloned into the NdeI and XhoI restriction sites of pET28b.

Site-direct mutagenesis of PA4781 (E314A mutant) was carried out by means of Quik change (Stratagene) kit starting from the pET-PA4781 template.

### Determination of intracellular c-di-GMP content

The *E. coli* strain (AB1548: AB472∆yhjH) lacking the endogenous PDE *yhjH* (a kind gift of Dr. A. Boehm), was used to perform the PA4108, PA4781 and PA4781_HD-GYP_ complementation assays. This strain presents higher levels of c-di-GMP than the control strain (AB472), due to the lack of the *yhjH* PDE; the control strain, used as reference of c-di-GMP levels, is a derivative of K-12 MG1655 with λDE3 (T7pol+) [31]. pET28b vector was used as negative control.

Cultures were grown at 37°C in Luria-Bertani (LB) medium; 0.1 mM IPTG (isopropyl β-d-thiogalactoside) was added at OD_600_ = 0.8 and cells were harvested by centrifugation after 2 hours of further growth. Overexpression of the proteins was verified by Western blot analysis with antisera against the His6-tagged tail (Santa Cruz, data not shown). Soluble cell extracts were prepared as previously described [32] and the nucleotide content was analysed by Reverse Phase HPLC (RP-HPLC) using a 150 x 4.6 mm reverse phase column (Prevail C8, Grace Davison Discovery Science) at room temperature on a LabFlow 4000 apparatus (LabService Analytica). Detection wavelength was 252 nm and the mobile phase was 100 mM phosphate buffer pH 5.8 / methanol (98/2, v/v). Data are the mean of at least two independent experiments, each being done in duplicate.

**Figure 1:**
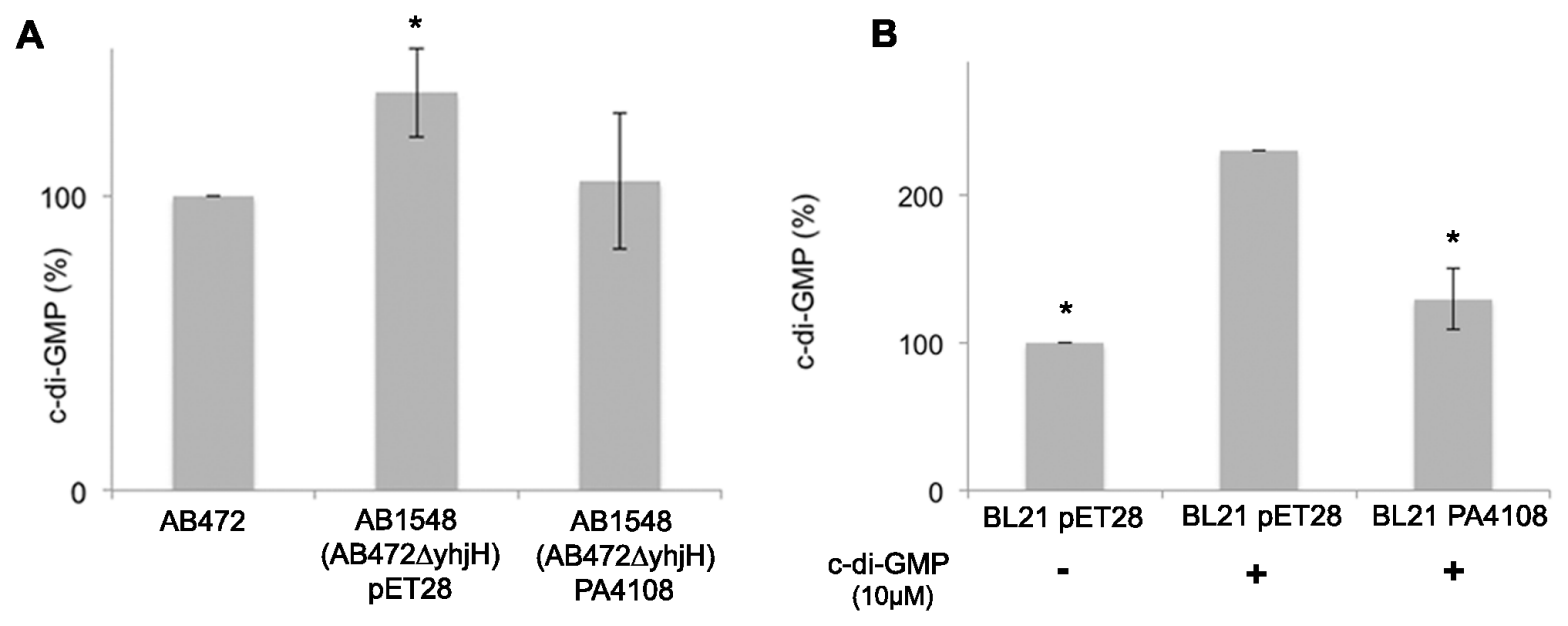
Effect of the overexpression of PA4108 on c-di-GMP levels in *E. coli.* (A) Comparison of c-di-GMP levels of the *E*. *coli* AB1548 mutant strain, lacking the endogenous PDE YhjH, containing only the pET28 plasmid (control strain) and those of the AB1548 strain complemented with PA4108 (AB1548(∆yhjH) PA4108 in the figure). c-di-GMP levels of the parental *E*. *coli* strain (AB472) were used as reference (100%). Data are the mean of at least two independent experiments, each being done in duplicate. (B) PDE activity in soluble cell extracts of BL21(DE3) *E*. *coli* strain overexpressing PA4108 (BL21 PA4108 in the figure), analysed after 3.5 h of incubation with 10 µM c-di-GMP. The control strain (BL21 pET28 in the figure) was analyzed after incubation with or without c-di-GMP, in order to evaluate the levels of unreacted c-di-GMP to be compared to the physiological levels of this background. Data are the mean of at least three independent experiments, each being done in duplicate. In (A) and (B) statistical significance with respect to *E*. *coli* AB472 or BL21 pET28 with c-di-GMP, respectively, is indicated with an asterisk (p < 0.01).

Statistical significance was calculated by ANOVA test with Bonferroni correction; a p-value ≤ 0.01 was considered statistically significant.

### Phosphodiesterase activity assay on soluble cell extracts

*E. coli* wild-type strain (BL21 (DE3)) overexpressing PA4108 or PA4781 or PA4781_HD-GYP_ was used to analyse the PDE activity of these proteins in soluble cell extracts. Cultures were grown in 25 ml of LB under constant shaking at 37°C until the cells reached late-exponential phase (OD_600_= 0.8-1.0). The *E. coli* strain containing the pET28b vector was grown under the same conditions, as a control. Cells were harvested by centrifugation and resuspended in 4 ml of PDE buffer (50 mM Tris-HCl pH 8.0, 10 mM MgCl_2_, 250 mM NaCl, 5 mM 2-mercaptoethanol, 1 mM PMSF, Complete EDTA-free protease inhibitor (Roche Diagnostics, Indianapolis)) and frozen overnight at -80°C, according to [3]. Cultures were thawed on ice and sonicated. Synthetic c-di-GMP (final concentration 10 µM, BIOLOG) was added to each lysate, and the reaction was allowed to proceed for 3.5 h at 30°C; control strain was also incubated with the same volume of water, under the same experimental conditions. The reaction was stopped by the addition of 2 ml of 1 M HClO_4_ and, after neutralization with 2 M K_2_CO_3_ each sample was centrifugated [3]. The supernatant was evaporated to dryness using a Speed Vac, resuspended in 500 µl of HPLC-running buffer (100mM sodium phosphate buffer pH 5.8), filtered with 2µm filters and analyzed by HPLC analysis, as described above. We performed at least two independent assays for each sample and the c-di-GMP peak in each sample was compared with that detected in the extract from the control strain, after incubation with c-di-GMP. Data are the mean of at least three independent experiments, each being done in duplicate. Statistical significance was calculated as described above.

**Figure 2:**
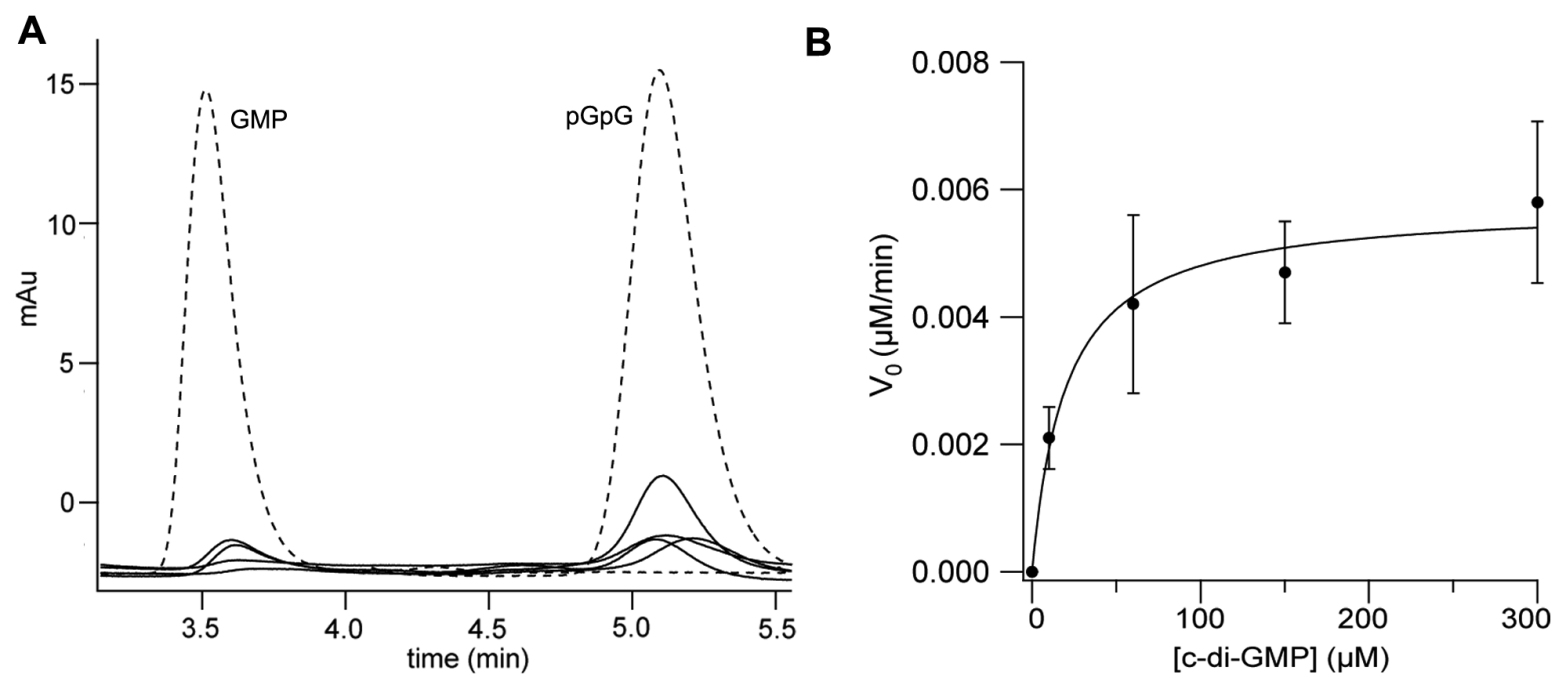
c-di-GMP hydrolytic activity of PA4108. (A) Representative time-courses of pGpG and GMP formation obtained after incubation of 0.64 µM PA4108 with 150 µM c-di-GMP (in the presence of 10 mM MgCl_2_ and 2.5 mM MnCl_2_). The nucleotide content of the reaction mixture was analyzed by RP-HPLC at different times (30, 60, 100, 180 min, at 30°C; solid lines in the figure) and compared with a calibration curve of standard solutions of GMP and pGpG (the chromatogram of a 5 µM solution of both nucleotides is reported as reference, dashed lines). (B) Plot of the initial rate of c-di-GMP hydrolysis (µM of c-di-GMP consumed/min) measured at different c-di-GMP concentrations (black circles). Data were fitted with the Michaelis-Menten equation (continuous line) in order to extrapolate the K_M_ and V_max_ parameters (20 µM and 5.8x10^-3^ µM/min, respectively). Data are the average of at least two independent experiments.

### Protein expression and purification

PA4781 (and its mutant E314A) and PA4781_HD-GYP_ expression was obtained after transformation of *E. coli* BL21 (DE3) strain. Overnight cultures were used to inoculate (1:100) 400 ml of liquid LB broth with Kanamycin (30mg/ml) and 0,1 mM IPTG. Cultures were grown under constant shaking at 37°C (full-length proteins) or 25°C (PA4781_HD-GYP_ mutant) until the cells reached late-exponential phase (OD_600_= 0,8-1,0). Cells were harvested by centrifugation and resuspended in lysis buffer (50mM TRIS pH 8, 50mM NaCl, 1mM PMSF).

The expression of the PA4108 protein was carried out in BL21plysS (DE3) *E. coli* strain under constant shaking at 37°C; protein expression was induced with 1 mM IPTG when OD_600_ was 0,7. Cells were harvested by centrifugation after 2h and the protein was purified as described below.

**Figure 3:**
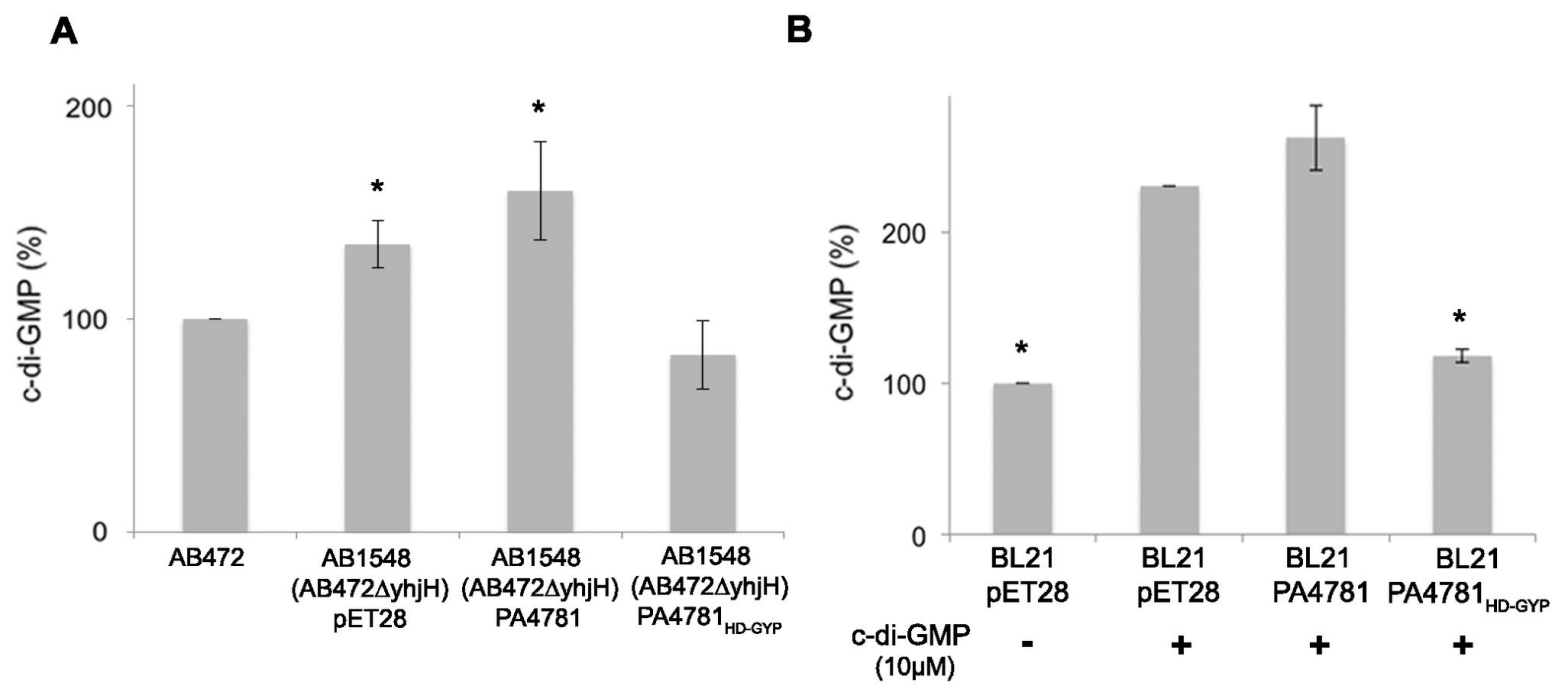
Effect of the overexpression of PA4781 on c-di-GMP levels in *E. coli*. (A) Comparison of c-di-GMP levels of the *E*. *coli* AB1548 mutant strain, lacking the endogenous PDE YhjH, containing only the pET28 plasmid (control strain) and those of the AB1548 strain complemented with either PA4781 or PA4781_HD-GYP_ (a truncated version of the protein lacking the REC domain). c-di-GMP levels of the parental *E*. *coli* strain (AB472) were used as reference (100%). Data are the mean of at least two independent experiments, each being done in duplicate. (B) PDE activity in soluble cell extracts of BL21(DE3) *E*. *coli* strain overexpressing either PA4781 or PA4781_HD-GYP_. The control strain (BL21 pET28 in the figure) was analysed after incubation with or without c-di-GMP, in order to evaluate the levels of unreacted c-di-GMP to be compared to the physiological levels in this background. Data are the mean of at least three independent experiments, each being done in duplicate. In (A) and (B) statistical significance with respect to *E*. *coli* AB472 or BL21 pET28 with c-di-GMP, respectively, is indicated with one asterisk (p < 0.01).

Cell lysis was carried out by ultrasonic treatment on ice and the cleared soluble extract was obtained by centrifugation. Proteins were purified as His-tagged fusions using a 5ml HisTrap column (GE Healtcare) equilibrated with 20 mM TRIS pH 7.6, 150 mM NaCl (buffer A). Elution has been carried out with a stepwise increase in imidazole concentration, with all proteins eluting at 200mM imidazole. The buffer exchange of pooled fractions was performed with PD-10 Desalting column (GE Healtcare).

The large part (70%) of the nucleotide content found in complex with purified PA4108 was removed by gel filtration (on a Superdex-200 column – GE-Healthcare) after incubation of the protein sample with 10 mM MgCl_2_ (for 1 hour).

The aggregation state of the proteins was analysed with a Superdex-75 10/30 column equilibrated with buffer A, using a HPLC apparatus (not shown). Extinction coefficients were calculated by means of the BCA (Sigma-Aldrich) assay and found to be 2, 0.59, 0.61 (mg/ml)^-1^cm^-1^, for PA4108, PA4781 and PA4781_HD-GYP_, respectively.

### Phosphodiesterase activity assay on purified proteins

#### Activation of PA4781 by acetyl phosphate

PA4781 (15 µM) was phosphorylated by incubation with 1000-fold molar excess of acetyl phosphate (e.g., 15 mM) in the phosphorylation buffer (50 mM Tris–HCl, 10 mM MgCl_2_, 0.1 mM EDTA, 0.1 mM DTT, and 0.1 M NaCl; pH 7.5) [33]. The phosphorylation reaction was carried out at 28°C for at least 20 min before substrate addition. Alternatively, PA4781 was incubated with beryllium fluoride (BeF_3_^-^) (not shown), a compound known to mimic the phosphoryl group [34]; however, this set up does not trigger REC domain activation.

#### c-di-GMP and pGpG hydrolysis

The ability of PA4108, PA4781 and PA4781_HD-GYP_ to hydrolyse c-di-GMP and, if indicated, pGpG, was assayed by incubation at 30°C in the reaction buffer (50 mM Tris–HCl pH 8, 150 mM NaCl) in the presence of 2.5 mM MnCl_2_, and/or 10 mM MgCl_2_ and excess of c-di-GMP or pGpG (BIOLOG) as substrate. The reaction was stopped in ice after different incubation times and the proteins were removed from the sample by filtration on 30000 Da MWCO concentrators at 4°C (Sartorius). This set up was chosen instead of the boiling procedure, since filtration of the reaction mixtures leads to higher recovery of nucleotides (higher specific activity but almost comparable K_M_ parameters, not shown). At least two independent assays for each sample were performed; the protein storage buffer was used as a negative control.

The K_M_ value was obtained by fitting the initial velocities obtained at various substrate concentrations with the Michaelis-Menten equation, using the Igor Pro software (Wavemetrics).

More in detail, the initial velocity of catalysis of PA4108 (0.64 µM) was measured at different c-di-GMP (10, 60, 150, 300 µM c-di-GMP) or pGpG (5, 10, 80, 150 µM pGpG) concentrations. The initial velocity of catalysis of PA4781 was measured at different c-di-GMP (30, 60, 120, 200, 300 µM c-di-GMP) or pGpG (2.5, 5, 10, 15, 25, 50, 80, 120 µM pGpG) concentrations, using pre-activated protein at a final concentration of 5 or 0,5 µM.

The initial velocity of catalysis of PA4781 E314A was measured at different c-di-GMP (10, 30, 60, 120, 200 µM c-di-GMP) or pGpG (2.5, 5, 10, 15, 25, 50, 80 µM pGpG) concentrations, using pre-activated protein at a final concentration of 5 or 0,5 µM.

The nucleotide content of the samples was measured by RP-HPLC analysis as described above. Synthetic c-di-GMP, pGpG (Biolog) and GMP (GE Healtcare) were also analysed under the same experimental conditions reported above.

### Modeling studies

Alignment of sequence data was performed using the multialign server (http://multalin.toulouse.inra.fr/multalin/) (multiple sequence alignment with hierarchical clustering [35]). Evolutionarily conserved residues were identified using the CAMPO server [36]. Predictions of secondary structure and intrinsically disordered regions were carried out as described previously [37]. Homology models of PA4781 and PA4108 were constructed by means of the modeller-9 package [38] and the PyMod interface [39], using the crystal structure of Bd1817 (sequence identity with PA4781: 32%; sequence identity with PA4108: 33%; PDB:3TM8; [28]). Ten different models were built and evaluated using several criteria: the model displaying the lowest objective function [40] was taken as the most representative and analyzed with ProsaII [41] and Verify_3d [42] to monitor its structural consistency. The initial alignment was then subjected to minor changes in an attempt to increase the low score regions, and resubmitted each time to a new modeller session. The final overall Verify_3d and prosaII plots showed a structure of good quality. The c-di-GMP moiety was then docked by means of autodock, version 4.2 (http://autodock.scripps.edu/), keeping all parameters at their default values [43].

## Results and Discussion

### PA4108 is a PDE which influences c-di-GMP levels in E. coli

The activity of PA4108 as a c-di-GMP-specific PDE was analysed by a complementation assay using a mutant strain (AB1548) of *E. coli* lacking the endogenous phosphodiesterase *yhjH*; AB1548 is characterized by higher levels of c-di-GMP than the control strain (AB472), which in turn was used as the reference for c-di-GMP levels.

The intracellular content of c-di-GMP was determined by RP-HPLC and the results are summarized in Figure 1A. The overexpression of PA4108 into the *E. coli* AB1548 mutant strain results in a significant decrease of c-di-GMP intracellular levels, thus restoring the levels observed in the control strain (AB472). This result confirms that PA4108 is active as a PDE, in agreement with the data obtained in *P. aeruginosa* [19], even in the *E. coli milieu*, which, as mentioned above, lacks HD-GYPs and possible regulatory pathway(s) specifically linked to HD-GYP PDEs. PA4108 contains an uncharacterized N-terminal domain (NTD), whose role in the regulation and mode of action of the protein is still unknown; however, if this domain plays a role in controlling catalysis, its activation (if any) is still possible in *E. coli*.

To confirm this result, the enzymatic activity of PA4108 was also independently measured in a soluble cell extract of the BL21(DE3) *E. coli* strain overexpressing PA4108. Briefly, the cell extract was incubated with exogenous (synthetic) c-di-GMP for 4 hours (30°C); the c-di-GMP level at the end of the incubation time was evaluated by RP-HPLC (Figure 1B). In agreement with the complementation assay described above, the residual c-di-GMP observed in the cell extract containing PA4108 was comparable to that of the sample lacking exogenous c-di-GMP, suggesting that the presence of PA4108 triggers the consumption of the exogenous c-di-GMP.

It should be mentioned that the expression of PA4108 (induced by adding IPTG from the beginning of the growth) significantly slows down the growth rate of *E. coli* (see Figure S1); this suggests that PA4108 PDE activity may alter and/or impair cell-cycle progression. The observed effect of PDE activity (and c-di-GMP levels) on the growth profile is in agreement with recent evidences showing that the fine-tuning of c-di-GMP levels mediates intracellular events that co-ordinately drive bacterial cell-cycle progression and development [44].

### PA4108 is able to degrade c-di-GMP in vitro

In order to shed light on the catalytic mechanism employed by this HD-GYP, the PA4108 protein was characterized biochemically. The purified protein is monomeric, as assayed by SEC (not shown) and no other species or aggregates are populated; at this stage, we do not know whether all HD-GYP PDEs share the same aggregation state and if this could be a relevant parameter in controlling catalysis. The sole HD-GYP structurally characterized to date is the unconventional HD-GYP protein from *Bdellovibrio* , which crystallized as a monomer [28].

Given that PDE activity is known to be metal-dependent [23,24], the enzymatic assay was initially optimized by exploring different metals and pH values (7.5-9, not shown). The optimal reaction conditions are: pH 8 at 30°C, in the presence of both Mg^2+^ and Mn^2+^; the specific activity, obtained from the initial rate of reaction with 150 µM c-di-GMP (Figure 2A), is 0.4 nmol c-di-GMP hydrolyzed /mg of protein/min. Our results represent the first characterization of the PDE activity of PA4108; it should be noticed that, even after 3 hours of incubation with the substrate, the PDE activity of PA4108 does not lead to relevant substrate consumption *in vitro*, contrary to previously characterized PDEs [20,22,45], being the turnover strikingly slow (see Table 1 for comparison).

**Table 1:** Kinetic parameters of the c-di-GMP specific phosphodiesterases characterized to date (belonging to the HD-GYP or the EAL subtype).

**PDE**	**Catalytic domain**	**K_M_ (µM**)	**k_cat_ (s^-1^**)	**Ref.**
PA4108	HD-GYP	20±5 (30±9)	1.5±0.1x10^-4^(1.2±0.1x10^-3^)	this work
PA4781^a^	HD-GYP	119±30 (27±8)	2.0±0.3x10^-4^(7.7±1x10^-4^)	this work
PA4781 E314A^a^	HD-GYP	6.8±2 (17±8)	6.0±1x10^-4^(2.0±0.3x10^-4^)	this work
PdeB (*Borrelia burgdorferi*)	HD-GYP	0.0029	n.d.	[22]
CC3396 (*Caulobacter crescentus*)	EAL	>100 0.24^a^	n.d.	[23]
RocR (*Pseudomonas aeruginosa*)	EAL	3.2	0.67	[46]
PA2567 (*Pseudomonas aeruginosa*)	EAL	5.2	0.39	[58]
VieA (*Vibrio cholerae*)	EAL	0.06	n.d.	[59]
BlrP1 (*Klebsiella pneumoniae*)	EAL	n.d.	0.13 0.54^a^	[54]

The values obtained using pGpG as a substrate are also indicated in brackets.

^a^ Activated

As expected for HD-GYP PDEs, hydrolysis of c-di-GMP produce GMP, contrary to the EAL sub-type of PDEs, where the reaction product is pGpG [20,22,46]; in addition, in the reaction catalysed by PA4108, a significant amount of pGpG also accumulated (Figure 2A). The capability of HD-GYP PDEs to populate pGpG as an intermediate, previously hypothesized but so far not clearly demonstrated [7], indicates that a two-step mechanism is employed during catalysis, being each phosphodiester bond hydrolyzed independently.

The K_M_ for c-di-GMP was determined by analysing the initial rate of c-di-GMP hydrolysis at different substrate concentrations (e.g. 10, 60, 150, 300 µM c-di-GMP) (Figure 2B). These experiments revealed that the K_M_ for c-di-GMP is rather high (~20 µM) compared to the values measured with other catalytically active enzymes such as the HD-GYP PdeB from *B. burgdorferi* (K_M_= 2.9 nM [22]) or other EAL-type PDEs (K_M_ from 60 nM to a few micromolar) (Table 1). It cannot be ruled out that the very slow kinetics observed has physiological relevance and that this protein works primarily as a c-di-GMP sensor (i.e. binding of c-di-GMP is not followed by catalysis), rather than a PDE.

**Figure 4:**
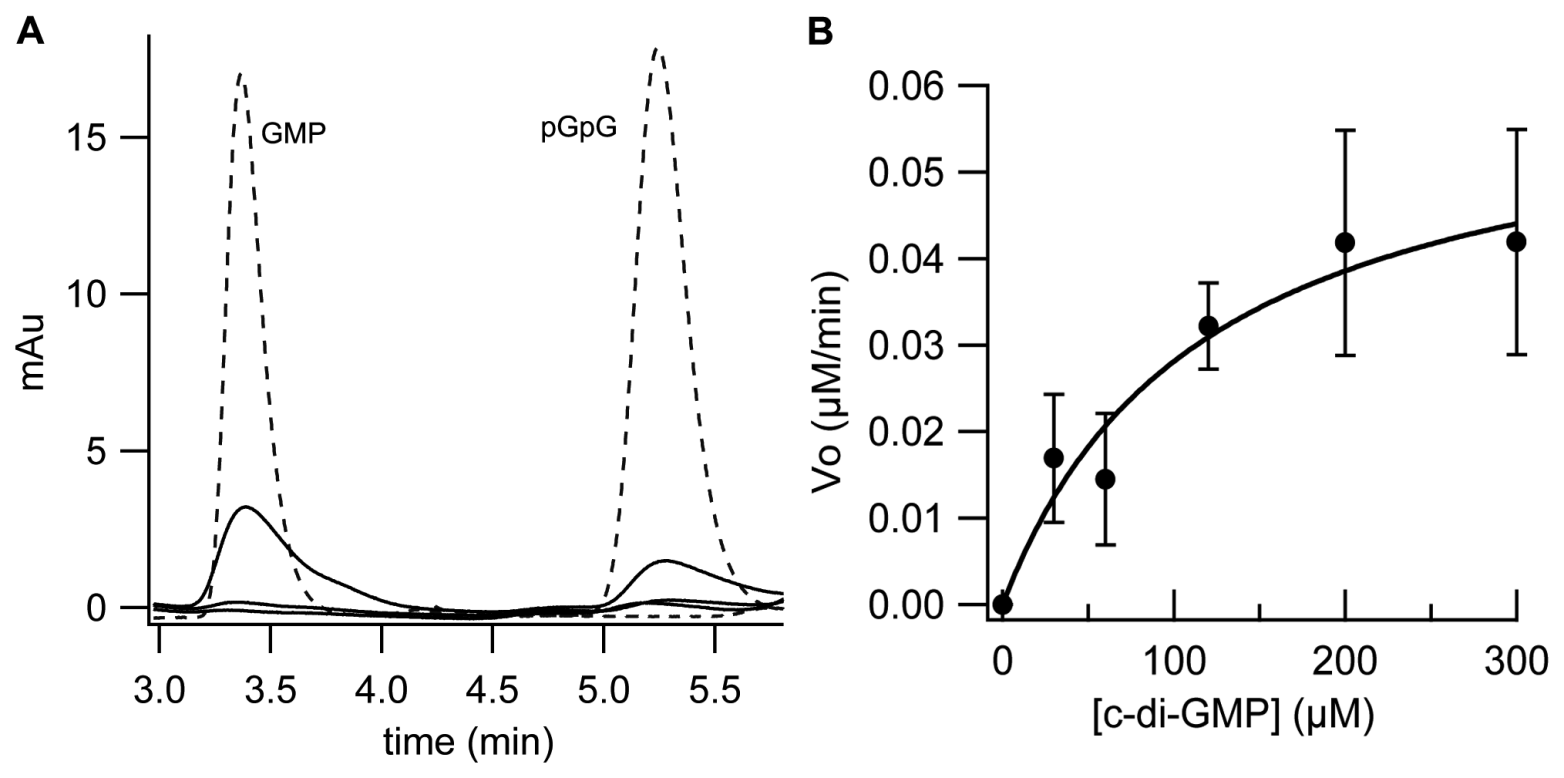
c-di-GMP hydrolytic activity of PA4781. (A) Representative time-courses of pGpG and GMP formation obtained after incubation of 5 µM phosphorylated PA4781 with 120 µM c-di-GMP (in the presence of 10 mM MgCl_2_). The nucleotide content of the reaction mixture was analyzed by RP-HPLC at different times (5, 10, 15 min, at 30°C; solid lines in the figure) and compared with a calibration curve of standard solutions of GMP and pGpG (the chromatogram of a 5 µM solution of both nucleotides is reported as reference, dashed lines). (B) Plot of the initial rate of c-di-GMP hydrolysis (µM of c-di-GMP consumed/min) measured at different c-di-GMP concentrations (black circles). Data were fitted with the Michaelis-Menten equation (K_M_ ~120 µM; V_max_=6x10^-2^ µM/min). Data are the average of at least two independent experiments.

During the purification procedure, we found a subpopulation of purified PA4108 protein in complex with nucleotide species, as it is evident in the UV spectrum below 270 nm (Figure S2, bold line). The presence of bound nucleotide(s) is a feature already highlighted in other purified proteins involved in c-di-GMP turnover [34,47]; as an example, PleD was found saturated with c-di-GMP, a physiological high affinity ligand (K_D_=0.3 µM) which is removed only after prolonged dialysis and SEC chromatography [34]. The nucleotides found in PA4108, analyzed by RP-HPLC, include both the substrate (c-di-GMP) and the intermediate pGpG; the amount of bound nucleotides was indeed very variable among different batches of purified protein (50-100%). A large part of these species (~70%) was removed by gel filtration after incubation with MgCl_2_.

This evidence suggests that, in the cellular *milieu*, the protein populates a significant fraction able to bind nucleotide(s) with high affinity; this heterogeneity cannot be easily interpreted, since it might be simply due to protein overexpression or it may originate from a more complex pattern, including partial activation of the NTD domain or interaction with possible partners *in vivo*, as shown for the HD-GYP archetype RpfG [21,30]. More in general, protein-protein interactions do play a key role in regulating different cellular functions connected to c-di-GMP metabolism in several bacteria [44,48,49,50].

### *E. coli* fails to activate the REC domain of PA4781 PDE

Contrary to expectations, the overexpression of the PA4781 protein neither decreases the c-di-GMP levels in *E. coli* (both intracellular or exogenous) (Figure 3) nor alters the growth profile (Figure S1), as compared to the control strains.

PA4781 harbours a HD-GYP catalytic domain and a CheY-like regulatory domain (a REC-domain belonging to response regulators, RR) located at the N-terminus of the protein. The *in vivo* results indicate that the REC domain of PA4781 cannot be allosterically activated by phosphorylation in the *E. coli* background, suggesting that a dedicated kinase is required, which might be absent in *E. coli*. According to this interpretation, no PDE activity was observed *in vitro* after incubation of purified PA4781 with c-di-GMP under different conditions (metals and pH) (Figure S3). The RR proteins found in the two-component regulators represent a versatile strategy to enable numerous regulatory mechanisms controlled by phosphorylation [51]. In order to trigger activation of the REC domain *in vitro*, PA4781 was phosphorylated with excess acetyl phosphate [33]. This setting restored the PDE activity of PA4781 (Figure 4A), thus confirming that activation of the REC domain *via* phosphorylation is necessary to switch PA4781 into an active form. Moreover this evidence confirms that the lack of activity in *E. coli* is due to the inability of this cellular environment to trigger REC domain activation.

The K_M_ and V_max_ values for PA4781 were determined as reported above for PA4108. Since under the experimental conditions employed, we cannot easily discriminate between the two forms (phosphorylated and non-phosphorylated proteins are both dimeric, data not shown) and thus we cannot quantify the amount of phosphorylated protein, we will refer to "apparent" K_M_ and V_max_, due to the uncertainty in the amount of phosphorylated protein. The activity at different c-di-GMP concentrations reveals that the apparent K_M_ is strikingly high (~120 µM) and the reaction proceeds remarkably slowly (specific activity of 0.28 nmol c-di-GMP hydrolyzed /mg of protein/min at 300 µM c-di-GMP) (Figure 4B and Table 1). At this stage we cannot assess whether the catalytic efficiency of PA4781 is intrinsically low or if it is due to experimental conditions; however, it is clear that understanding the mechanism employed by the REC domain in controlling the activity of PA4781 is mandatory for further characterization of this HD-GYP.

### The REC domain of PA4781 masks the HD-GYP catalytic domain

The mechanism whereby the nonphosphorylated REC domain negatively controls the PDE activity of PA4781 has been investigated more deeply. Contrary to PA4108, the UV spectrum of PA4781 displays a single peak at 280 nm which suggests that the protein is purified free of nucleotide species (Figure S2, thin line). The inability of the protein to bind c-di-GMP with high affinity was confirmed by ITC experiments: the titration profile of PA4781 with c-di-GMP superimposes with that of the control experiment (i.e. c-di-GMP diluted into the buffer solution), indicating that no significant binding of c-di-GMP to PA4781 has occurred (see Figure S4).

It is likely that the nonphosphorylated REC domain traps the protein in an inactive state, which hampers c-di-GMP binding. Phosphorylation of the REC domain may trigger the conformational change necessary to unmask the active site, thus allowing PA4781 to bind c-di-GMP and enter a catalytic cycle. To prove this hypothesis, a truncated version of the protein lacking the entire REC domain was produced, by removing the first 111 residues (hereinafter PA4781_HD-GYP_). Contrary to the full-length counterpart, the overexpression of PA4781_HD-GYP_ decreases the c-di-GMP levels in *E. coli* (both intracellular or exogenous) (Figure 3) and slows down the growth rate (Figure S1), as compared to the control strain. These results indicate that the isolated catalytic domain of PA4781 is constitutively active in *E. coli*.

UV spectra of the PA4781_HD-GYP_ purified protein (Figure S2) reveals that a significant fraction of the protein binds nucleotides with high affinity (as observed for PA4108) suggesting that, after removal of the REC domain, the HD-GYP domain is able to bind nucleotides. The PA4781_HD-GYP_ protein is mainly a monomer, as determined by gel filtration (data not shown), contrary to the full-length protein, and displays little, but significant PDE activity (~5 folds lower than that of phosphorylated PA4781, not shown). It is likely that RR-containing effectors are catalytically competent as dimers (as observed for the phosphorylated full-length protein) rather than as monomers. The lower PDE activity *in vitro* of PA4781_HD-GYP_ may thus represent the residual activity of this non-physiological aggregation state. Since this protein sample tends to aggregate and precipitate upon purification, it is likely that the active enzyme has been overestimated in the kinetic assays.

To probe the hypothesis that the REC domain may mask the HD-GYP domain, we have investigated the accessibility of the HD-GYP domain in the PA4781 and PA4781_HD-GYP_ proteins by limited proteolysis. Bioinformatic analysis indicates that the PA4781 sequence contains at least one proteolytic site for thrombin (residue 288) at the level of the loop containing the GYP signature (retained in the PA4781_HD-GYP_ construct). As shown in Figure S5, the full-length PA4781 sample is resistant to proteolysis, while PA4781_HD-GYP_ is readily degraded. This evidence clearly indicates that the HD-GYP domain of the two proteins is differently exposed to the solvent (and thus to thrombin), being that of the full-length protein masked by the presence of the REC domain (and thus resistant to proteolysis).

In summary, we can propose that the unphosphorylated REC domain acts as a lid of the HD-GYP catalytic domain, which hampers substrate binding. Upon phosphorylation, a rearrangement of the REC domain takes place thus enabling access to the HD-GYP active site and consequently substrate binding and hydrolysis [52].

**Figure 5:**
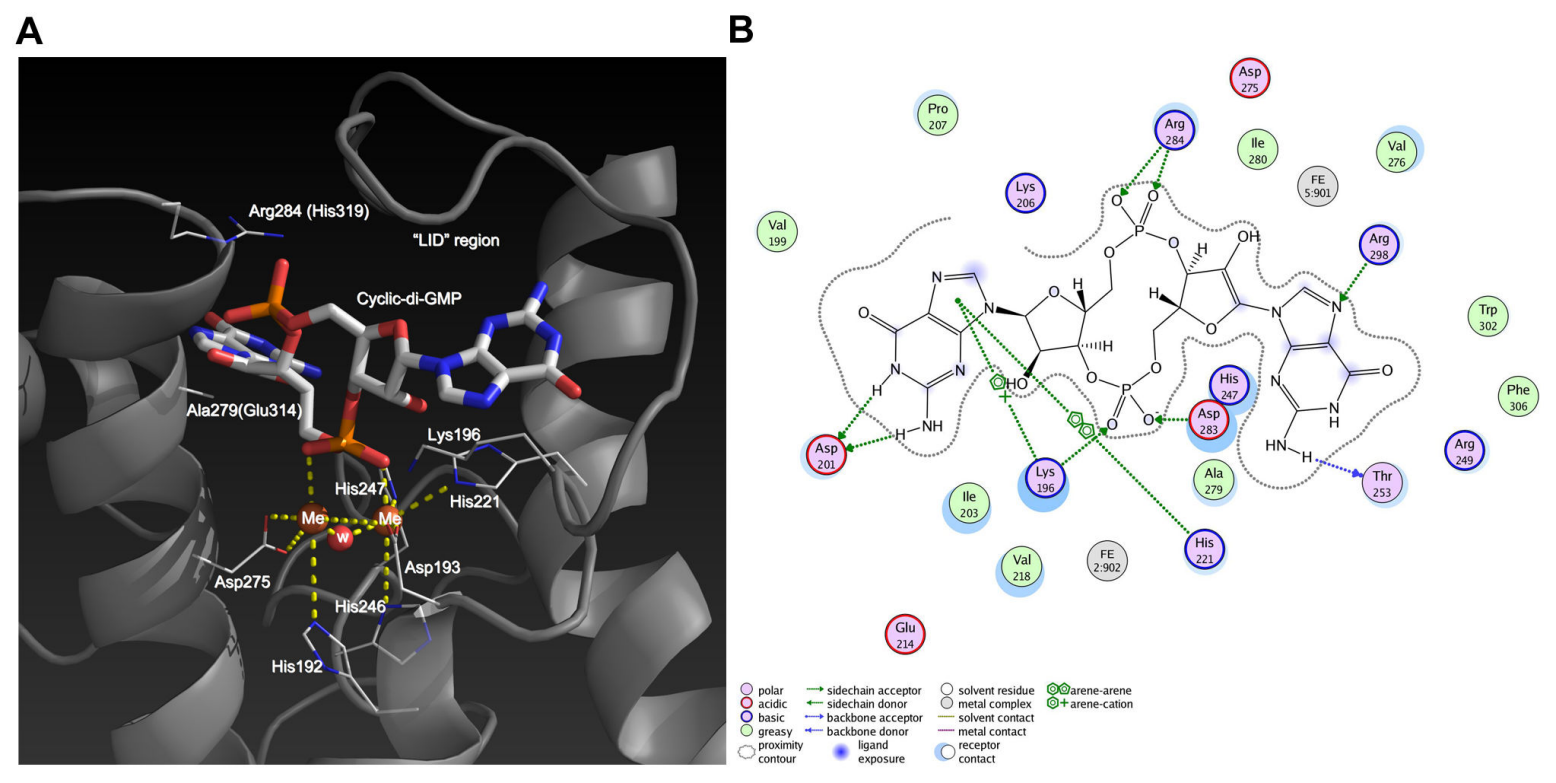
Predicted interaction of c-di-GMP with PA4108. (A) Model of the active site of PA4108 in complex with c-di-GMP. Residues in parenthesis indicate amino acid substitutions observed in PA4781. (B) The interaction of PA4108 with the substrate is represented.

### Structural determinants of substrate specificity in PA4781 and PA4108

The enzymatic characterization of PA4108 and PA4781 (reported above) indicates that, *in vitro*, hydrolysis of c-di-GMP is remarkably slow. It is indeed recently reported that the HD-GYP protein homologous Bd1817 from the predatory bacterium *B* *. bacteriovorus* [28] is completely inactive as a PDE. In the latter case, the lack of any detectable PDE activity or c-di-GMP binding *in vitro* was related to the non-consensus nature of several active-site residues of this HD-GYP protein. Therefore, we speculated whether a similar situation might occur in PA4108 and PA4781. To this end, we modelled the structure of the two HD-GYP proteins, using as template the crystal structure of Bd1817 (PDB: 3TM8). The residues coordinating the binuclear metal motif that is typical of HD-GYP proteins are invariant, with the only exception of Glu238 and Asn265 (sequence numbering refers to Bd1817), which are replaced in PA4108 and PA4781 by conserved His and Asp residues, respectively (His247 and Asp275 for PA4108; His282 and Asp310 for PA4781; Figure 5). His and Asp residues represent the canonical residues at such positions, as previously highlighted in a large-scale alignment of HD-GYP sequences [53]. Indeed, as already indicated by Lovering et al. [28], an aspartate residue equivalent to Asn265 would be able to perform acid-base catalysis, by activating the hydroxide anion that is responsible for the nucleophilic attack on the phosphorus atom of c-di-GMP. A consensus Lys residue at the HDxxK position, which is conserved in PA4781 (Lys224), PA4108 (Lys196) and most of the HD-GYP sequences, with its long side chain, could be responsible for the protonation of the leaving group, via a water-acidic residue relay [27,28,54]. This mechanism, involving a residue one helical turn on from the HD motif, has been already observed in other HD proteins (e.g., HDxxE^72^ in YfbR, where the E72A mutant correlates with no detectable activity [55]).

Another non-consensus residue of Bd1817, Glu274, is replaced in PA4108 (but not in PA4781) by a consensus arginine (Arg284), whose conservation and position has been already predicted to be necessary for the complexation with the second phosphodiester group of c-di-GMP. In PA4781, the consensus arginine is replaced by a His residue (His319; Figure 5). The presence of a His residue at such position could be coupled, in PA4781, to another non-canonical substitution, involving a conserved Ala residue (Ala279 in PA4108) in the c-di-GMP binding site (which is represented by the noncanonical Arg269 in Bd1817, and Glu314 of PA4781). The evolutionary conservation of Ala at such position is probably required to provide the necessary room to accommodate c-di-GMP. Indeed, modelling the c-di-GMP substrate into the binding cleft of PA4781 predicts that Glu314, upon interaction with His319, would generate a steric clash with the distal phosphodiester group of c-di-GMP. The presence of the topologically equivalent Arg269 in Bd1817 has been indicated as a possible reason why no c-di-GMP binding was observed [28].

In conclusion, the steric hindrance between Glu314 and the distal phosphodiester moiety does not allow modelling the interaction of c-di-GMP with PA4781. However, it cannot be ruled out that a conformational transition in response to an appropriate stimulus (such as REC domain phosphorylation) could trigger the activation of the HD-GYP domain.

**Figure 6:**
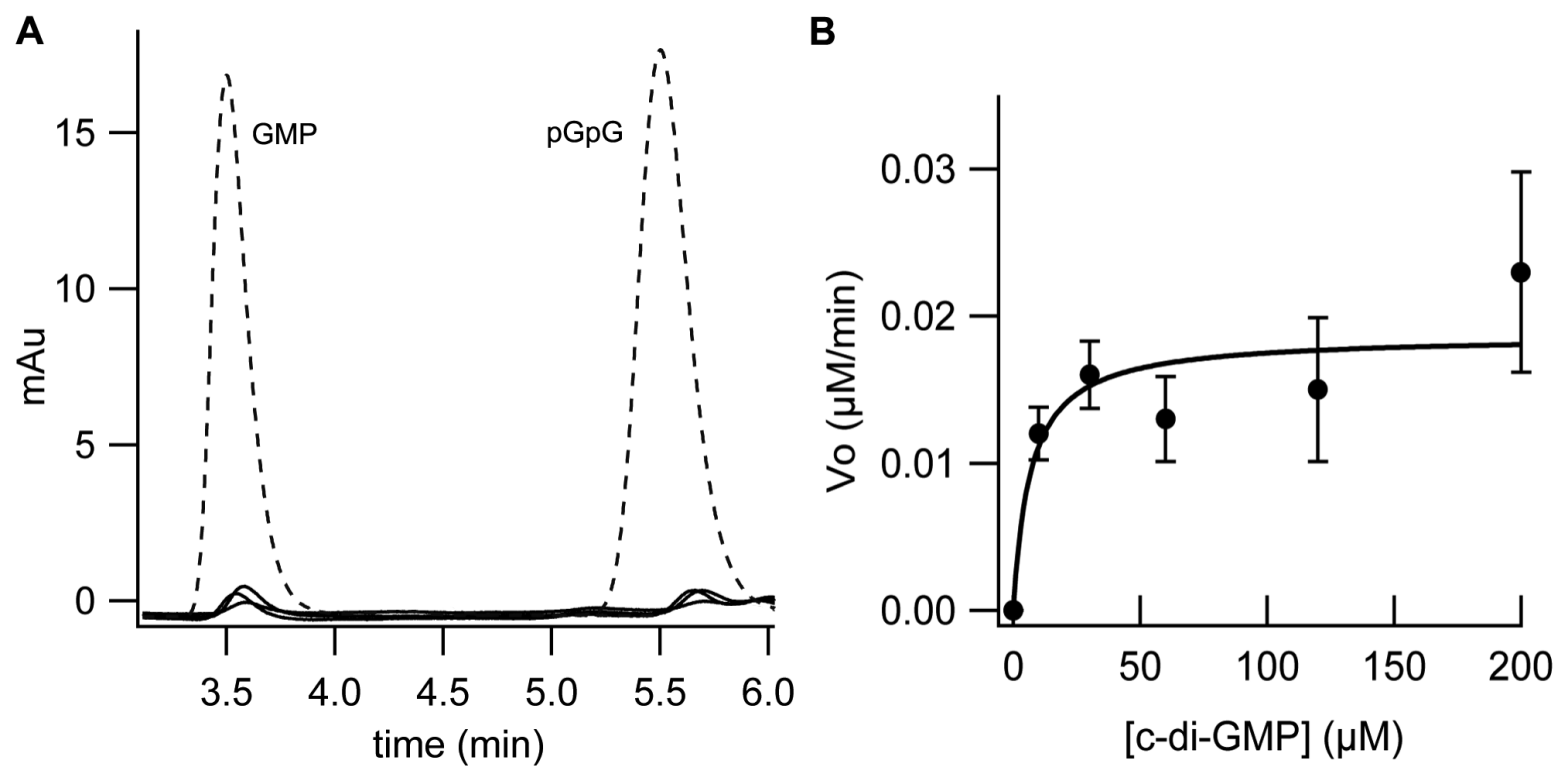
c-di-GMP hydrolytic activity of the PA4781 E314A mutant. (A) Representative time-courses of pGpG and GMP formation obtained after incubation of 5 µM phosphorylated PA4781 E314A with 10 µM c-di-GMP (in the presence of 10 mM MgCl_2_); the nucleotide content of the reaction mixture was analyzed by RP-HPLC at different times (5, 10, 15 min, at 30°C; solid lines in the figure) and compared with a calibration curve of standard solutions of GMP and pGpG (the chromatogram of a 5 µM solution of both nucleotides is reported as reference, dashed lines). (B) Plot of the initial rate of c-di-GMP hydrolysis (µM of c-di-GMP consumed/min) measured at different c-di-GMP concentrations (black circles). Data were fitted with the Michaelis-Menten equation (K_M_ ~7 µM; V_max_=1.9x10^-2^ µM/min). Data are the average of at least two independent experiments.

On the other hand, modelling the c-di-GMP substrate into the binding cleft of PA4108 was possible; the docking of c-di-GMP in the binding site of PA4108 required a structural rearrangement of the “lid” region, leaving the substrate buried apart from the solvent (Figure 5). The need of small rearrangements of several residues in the “lid” region of the HD-GYP domain to allow an expansion of the active site cleft was already discussed for Bd1817 [28]. Briefly, we predict that PA4108 interacts and accommodates c-di-GMP into a shallow binding groove, where a number of favourable interactions take place: the guanidine rings of c-di-GMP are stabilized by hydrogen-bonding interactions with Thr253, Arg269 and Asp201, and by arene-arene and arene-cation interactions with His221 and Lys196. The latter residue is also involved in an ion-pair interaction with a phosphate moiety of c-di-GMP. The other phosphate moiety is mainly stabilized by ion-pair interactions with Arg284.

### PA4781 and pGpG

The bioinformatic analysis reported above suggests that the predicted catalytic site of PA4781 is not optimal to locate c-di-GMP (compared to PA4108), mainly due to the steric hindrance between Glu314 and the distal phosphodiester moiety of c-di-GMP.

**Figure 7:**
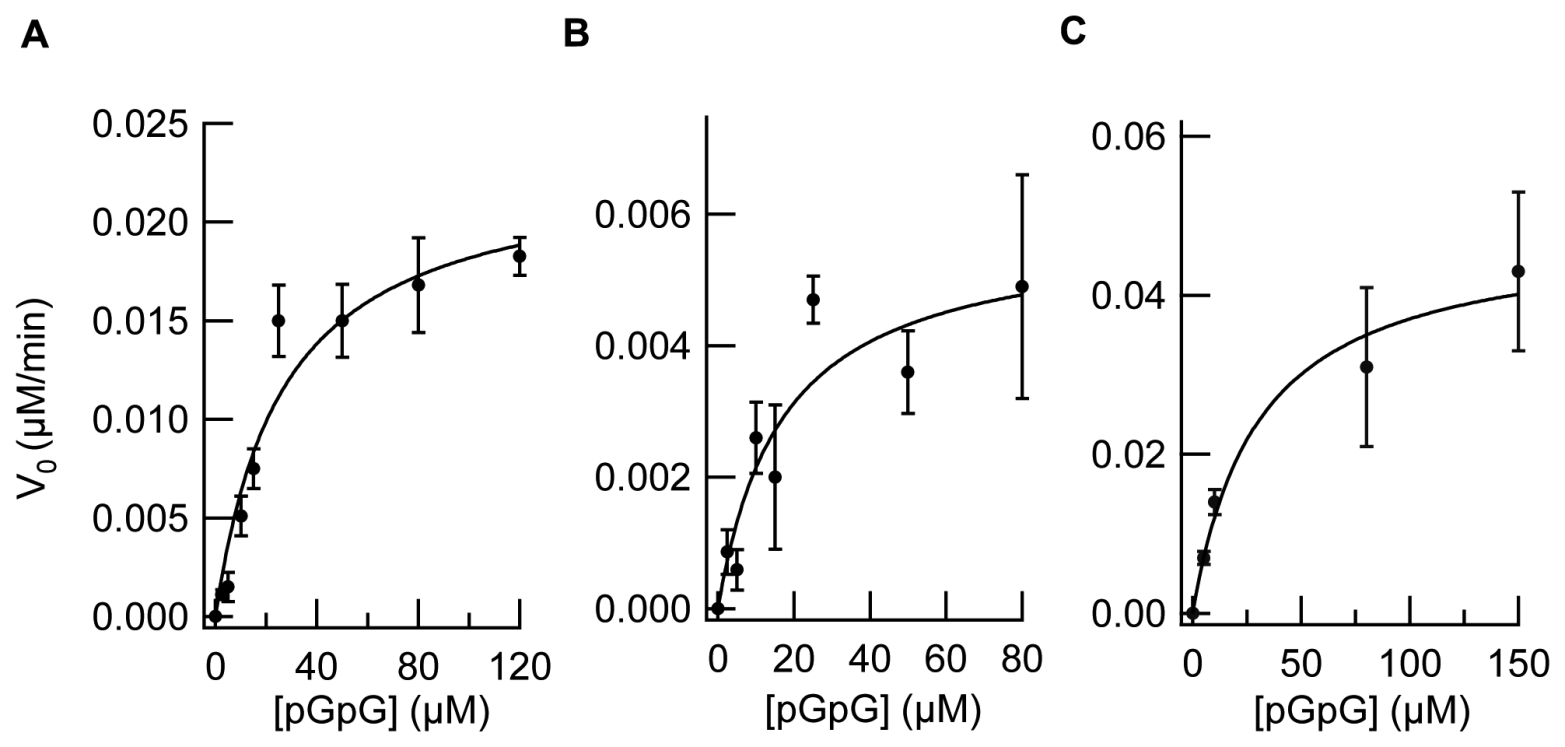
pGpG hydrolytic activity of (A) PA4781, (B) PA4781 E314A, (C) PA4108. For each protein, the initial rate of pGpG hydrolysis (µM of pGpG consumed/min) measured at different pGpG concentrations (black circles) was plotted as a function of µM pGpG. Data were fitted with the Michaelis-Menten equation (continuous line). It should be mentioned that the K_M_ of PA4108 for pGpG could be an “apparent” value due to the presence of ^~^30% of c-di-GMP still bound to the protein. Data are the average of at least two independent experiments.

In order to prove this hypothesis, the nonconsensus Glu314 of PA4781 was mutated into the consensus residue Ala (E314A) and the kinetic behaviour of the corresponding mutant (phosphorylated) was characterized (Figure 6A). The K_M_ for c-di-GMP was found to be ~7 µM and the V_max_~1,9x10^-2^ µM/min (Figure 6B). In agreement with bioinformatics predictions, the affinity for c-di-GMP of E314A is strikingly higher as compared to the wild-type counterpart (~17 folds), thus confirming that the active site of PA4781 is not designed to bind c-di-GMP, due to the presence of Glu314. On the other hand, the linear derivative (i.e. pGpG) does not present the ring constrain of c-di-GMP; it is likely that, considering the *in silico* studies and their *in vitro* validation (i.e. the E314A mutant), PA4781 binds pGpG with higher affinity than c-di-GMP.Therefore, the hydrolytic activity of PA4781 (pre-activated with acetyl phosphate) was assayed using pGpG as a substrate in the absence of c-di-GMP; a small fraction of pGpG was indeed converted into GMP, thus demonstrating that PA4781 displays a pGpG hydrolase activity. Surprisingly, despite the slow kinetics of the reaction (Vmax~2x10^-2^ µM/min), PA4781 displays a remarkably higher affinity for pGpG (K_M_~27 µM, Figure 7A) than for c-di-GMP. In light of these results, the K_M_ for pGpG of the PA4781 E314A mutant and of PA4108 was also measured and found to be ~17 µM and ~30 µM, respectively (Figure 7B and C) (see also Table 1). In such a context, it is likely that the capability to hydrolyse pGpG into GMP is a general feature of the HD-GYP proteins; moreover, the presence of the consensus Ala is crucial to bind c-di-GMP with “high” affinity in both enzymes, but it does not significantly perturb the affinity for pGpG.

Unfortunately, the intrinsic capability of both PA4781 or PA4108 to hydrolyze pGpG *in vivo* or in cellular extracts cannot be evaluated, being pGpG also hydrolysed by nonspecific hydrolases in *E. coli* [7]. Therefore, we cannot rule out that a possible biological role of a group of HD-GYP PDEs including PA4781, in the phosphorylated state, is to bind pGpG and possibly to hydrolyze it. If this is the case, we have to re-consider the current view of the role of pGpG in c-di-GMP metabolism.

## Conclusions

The involvement of the bacterial second messenger c-di-GMP in the control of biofilm formation and in the stimulation of the host immune system represents a hot topic among the scientific community due to the clinical relevance of biofilm-mediated infections. The number of research groups currently working on this attractive issue is rapidly growing and functional and structural data on the enzymes involved in c-di-GMP homeostasis start to be available. However, the requirement of biochemical data on the HD-GYP group of phosphodiesterases [7] has been very recently remarked, since their functional characterization is to date preliminary [21,30] and only one crystallographic structure is available [28].

The main aim of this paper was thus to improve our knowledge on this class of enzymes. This study is focused on two HD-GYP proteins from *Pseudomonas aeruginosa*, a model organism for the study of biofilms. These HD-GYP proteins, namely PA4108 and PA4781, were found to be involved in controlling biofilm formation *in vivo* [19].

Briefly, we show that: (i) PA4108 presents a very low (and slow) c-di-GMP hydrolytic activity *in vitro*, (ii) full-length PA4781 is not active in *E. coli* since this background fails to activate the REC domain, (iii) the REC domain of PA4781 masks the HD-GYP active site, (iv) the active site of PA4781 seems more suited to bind pGpG rather than c-di-GMP: the *in vitro* affinity of PA4781 for pGpG is ~5 fold higher than that for c-di-GMP, due to the presence of a nonconsensus glutamate (as proved by mutagenesis), (v) both PA4108 and PA4781 are able to hydrolyse pGpG into GMP. The functional characterization reported in this study indicates that the catalytic mechanism, the allosteric regulation and the regulatory network controlling both enzymes are more complex and even more puzzling than expected.

Concerning the catalytic mechanism, our biochemical data clearly show that pGpG is the intermediate species populated in the reaction of HD-GYP phosphodiesterases to yield GMP. The catalytic parameters indicate that the reaction proceeds slowly *in vitro*; we cannot exclude that full activation of catalysis is not achieved under our experimental conditions. Our data allow us to hypothesize that the RR-containing PA4781 co-evolved with a partner kinase, which specifically recognizes the REC domain linked to the HD-GYP one. Consequently, this partner kinase may be present in *P. aeruginosa* and in *X. campestris* [19], the latter containing the archetype HD-GYP RpfG, but not in *E. coli*, which does not present HD-GYP PDEs and seems unable to phosphorylate PA4781. The identification of this kinase is thus crucial and biochemical and bioinformatic studies are currently ongoing. On the other hand, while the mechanism of activation of PA4781 upon phosphorylation of the REC domain has been biochemically investigated in the present study, more data are needed to elucidate the role of the NTD in the activation of PA4108. Moreover, we cannot rule out that other macromolecular partner(s) are involved in controlling PA4781 and/or PA4108 activity, as previously demonstrated for other HD-GYP PDEs [7,21,29,30].

Our results also clearly reinforce the view that HD-GYP proteins, including *P. aeruginosa* PA4108 and PA4781, may not work solely as genuine PDEs, thus suggesting that the very low hydrolytic activity (see Table 1) may have physiological relevance. A possible role (alternative or secondary) of these proteins in sensing c-di-GMP and possibly pGpG cannot be excluded (this work and [7]). It is likely that both nucleotides acts as signal molecules (rather than as substrate/intermediate) for these HD-GYP PDEs, as already described for other c-di-GMP-dependent enzymes [7]. Given that the affinity for pGpG of PA4781 is higher than that for c-di-GMP, we can speculate that pGpG itself may act as a signal molecule (or a substrate), alternative to c-di-GMP. As underlined by Römling and coworkers [7], pGpG is indeed a member of "nanoRNA" molecules, which are known to be involved in controlling gene expression [56,57]. If this is the case, pGpG may accumulate once c-di-GMP reaches a threshold concentration and possibly exerts its role as a signal.

We feel that more data are needed in order to unveil whether this class of effectors acts as ancestral enzymes and how they are connected to the c-di-GMP-regulatory network; a complete picture will only be reached by coupling different experimental approaches, ranging from *in vitro* mechanistic studies to cell physiology.

## Supporting Information

Figure S1 **Bacterial growth curves.**
(PDF)

Figure S2 **UV spectra of purified HD-GYP proteins.**
(PDF)

Figure S3 **Catalytic assay on non-phosphorylated PA4781.**
(PDF)

Figure S4 **ITC experiments on PA4781.**
(PDF)

Figure S5 **SDS-PAGE of protein samples after limited proteolysis.**
(PDF)
