# Supplementary material for: C-di-GMP Hydrolysis by Pseudomonas aeruginosa HD-GYP Phosphodiesterases: Analysis of the Reaction Mechanism and Novel Roles for pGpG
Source: PLoS One. 2013 Sep 16;8(9):e74920. doi: 10.1371/journal.pone.0074920 (PMC3774798; doi:10.1371/journal.pone.0074920)
Supplement: Figure S1 — Bacterial growth curves. (PDF) [file pone.0074920.s001.pdf]

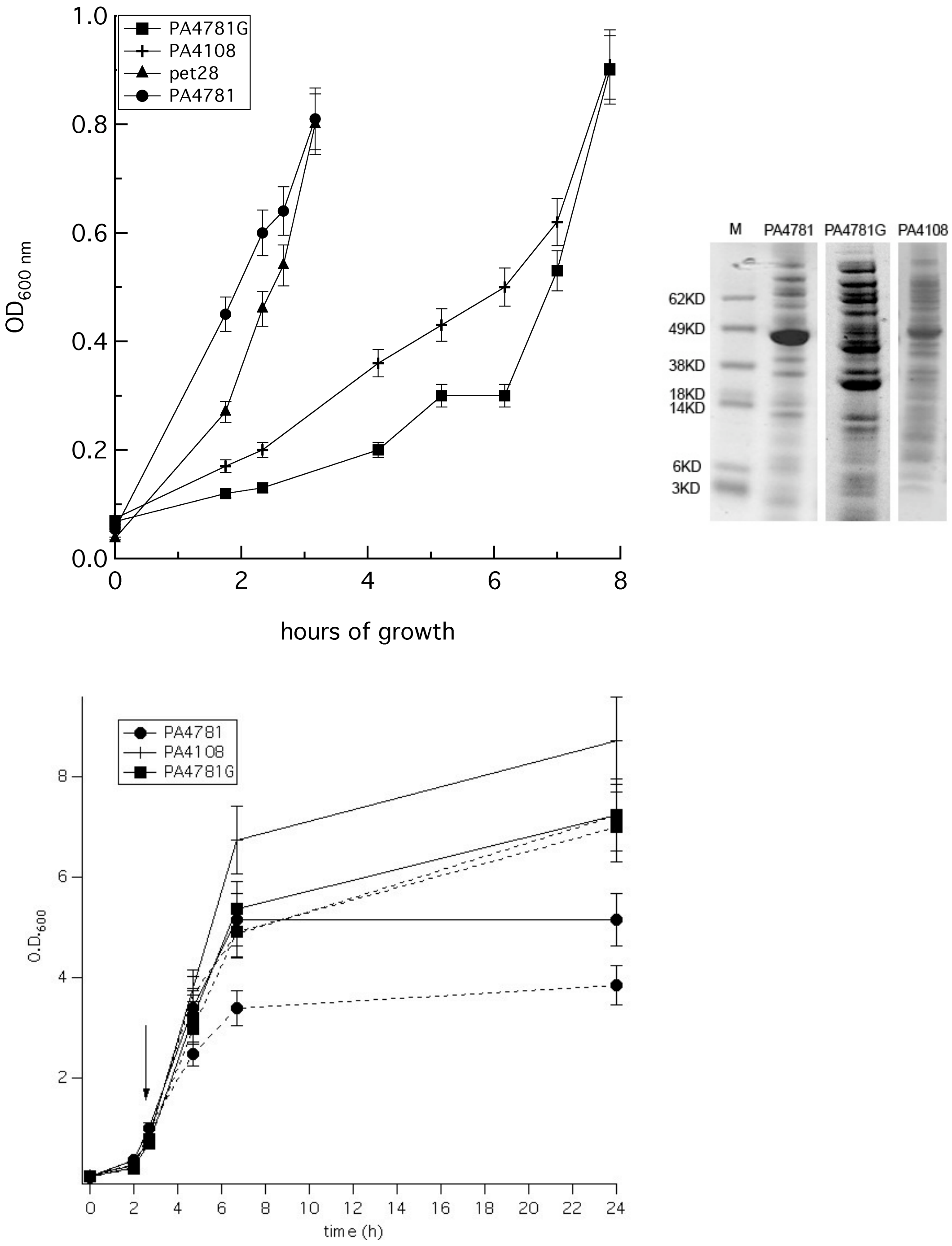

Figure S1. Growth curves of BL21(DE3) *E. coli* overexpressing the proteins reported in the legend. The strain containing the sole pET28 vector was used as a control.

Upper panel: Bacterial growth was followed in the presence of 0.1 mM IPTG from the beginning of the growth until OD600 reached 0.8-1. Protein expression levels (assayed by SDS-PAGE) are also included.

Lower panel: Bacterial growth was also followed after induction (dashed lines) of protein expression with 0.1 mM IPTG when OD600 was 0.7-0.8 (arrow). Control growths without IPTG addition were also analyzed (continous lines).
