# Supplementary material for: C-di-GMP Hydrolysis by Pseudomonas aeruginosa HD-GYP Phosphodiesterases: Analysis of the Reaction Mechanism and Novel Roles for pGpG
Source: PLoS One. 2013 Sep 16;8(9):e74920. doi: 10.1371/journal.pone.0074920 (PMC3774798; doi:10.1371/journal.pone.0074920)
Supplement: Figure S2 — UV spectra of purified HD-GYP proteins. (PDF) [file pone.0074920.s002.pdf]

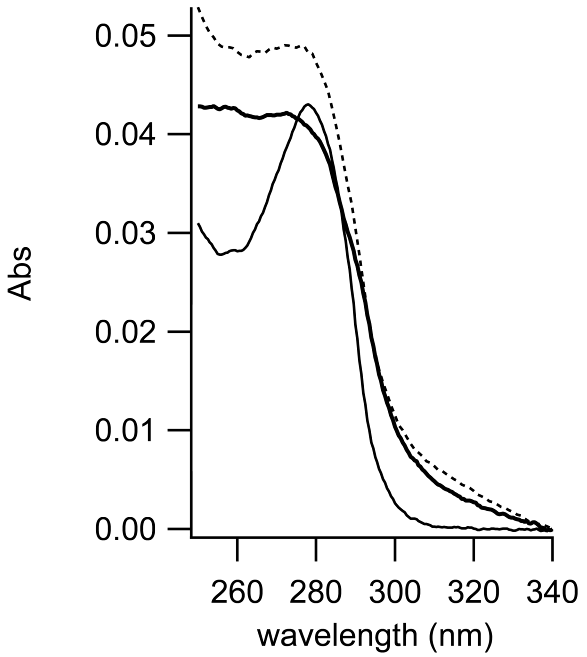

Figure S2. UV-spectra (250-340nm) of purified proteins characterized in the present study: PA4108 (bold line), PA4781 (nonphosphorylated, thin line) and PA4781$_{HD\text{-}GYP}$ (dotted line). The high absorption contribution below 270 nm found in PA4108 and PA4781$_{HD\text{-}GYP}$ spectra is due to the presence of nucleotide species bound to the proteins.
