# Supplementary material for: C-di-GMP Hydrolysis by Pseudomonas aeruginosa HD-GYP Phosphodiesterases: Analysis of the Reaction Mechanism and Novel Roles for pGpG
Source: PLoS One. 2013 Sep 16;8(9):e74920. doi: 10.1371/journal.pone.0074920 (PMC3774798; doi:10.1371/journal.pone.0074920)
Supplement: Figure S3 — Catalytic assay on non-phosphorylated PA4781. (PDF) [file pone.0074920.s003.pdf]

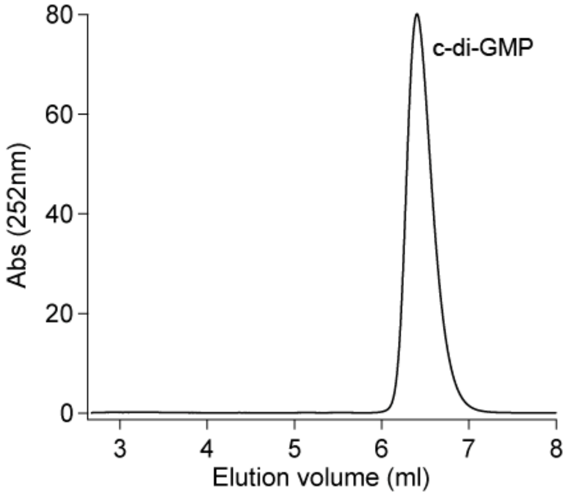

Figure S3. PA4781 catalytic assay. Reaction of 10 μM PA4781 with 30 μM c-di-GMP in the presence of 10 mM $MgCl_2$ and 2.5 mM $MnCl_2$ (4 hours, at 30°C); the nucleotide content of the reaction mixture was analyzed by RP-HPLC (bold line): no hydrolysis of c-di-GMP was observed.
