# Supplementary material for: C-di-GMP Hydrolysis by Pseudomonas aeruginosa HD-GYP Phosphodiesterases: Analysis of the Reaction Mechanism and Novel Roles for pGpG
Source: PLoS One. 2013 Sep 16;8(9):e74920. doi: 10.1371/journal.pone.0074920 (PMC3774798; doi:10.1371/journal.pone.0074920)
Supplement: Figure S4 — ITC experiments on PA4781. (PDF) [file pone.0074920.s004.pdf]

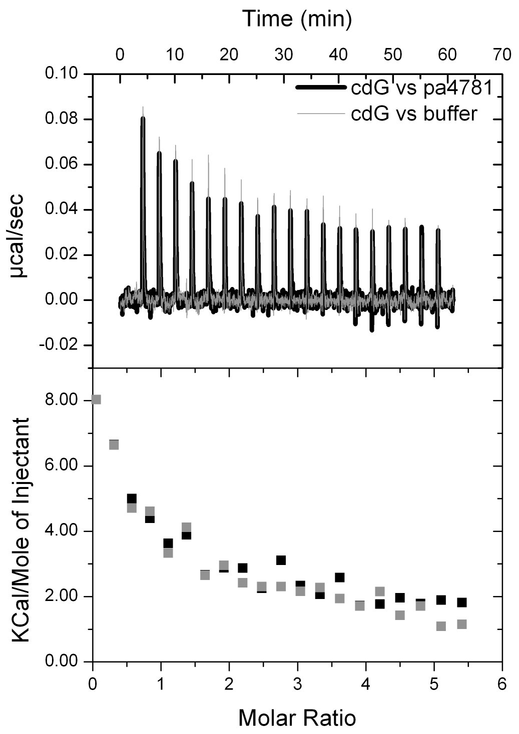

Figure S4. Binding of c-di-GMP to PA4781 was assayed by ITC using an iTC200 microcalorimeter (MicroCal). The PA4781 solution (3 µM) was prepared in 100 mM Tris (pH 8.5), 50 mM NaCl, 10 mM $MgCl_2$ and 2.5 mM $MnCl_2$. The c-di-GMP solution was prepared by dilution of a 1mM stock solution (in water) with the corresponding protein buffer. 2-µl aliquots of c-di-GMP solution (80 µM) were injected into the corresponding protein solution at 25°C. Upper panel: Raw ITC data of microcalorimetric titration of 3 µM PA4781 (nonphosphorylated) with 80 µM c-di-GMP (black trace). As control, a buffer solution was titrated with 80 µM c-di-GMP (grey trace). The two experiments are superimposable, indicating that no binding of c-di-GMP to PA4781 has occurred. As previously published [1], the heat exchange observed upon c-di-GMP dilution is related to the varying degree of dimerization of c-di-GMP in solution; integrated peak areas profile (lower panel, black and grey squares) could represent a dissociation equilibrium of the ligand [2]. When specific c-di-GMP/protein interaction does occur, as reported for PleD, the effect due to c-di-GMP dilution, if present, is limited to the first few injections and does not interfere with the sigmoidal part of the binding curve [1].

Titration of phosphorylated PA4781 with c-di-GMP could not be carried out, due to precipitation of calcium phosphate. Titration of PA4108 with c-di-GMP was attempted, even though protein precipitation and high c-di-GMP background signal yielded low quality data and a very low c-value (<0.05); therefore this experiment is not included and discussed in the present study.

1. Paul R, Abel S, Wassmann P, Beck A, Heerklotz H, et al. (2007) J Biol Chem 282: 29170-29177.
2. Heerklotz HH, Binder H, Epand RM (1999) Biophys J 76: 2606-2613.
