# Supplementary material for: C-di-GMP Hydrolysis by Pseudomonas aeruginosa HD-GYP Phosphodiesterases: Analysis of the Reaction Mechanism and Novel Roles for pGpG
Source: PLoS One. 2013 Sep 16;8(9):e74920. doi: 10.1371/journal.pone.0074920 (PMC3774798; doi:10.1371/journal.pone.0074920)
Supplement: Figure S5 — SDS-PAGE of protein samples after limited proteolysis. (PDF) [file pone.0074920.s005.pdf]

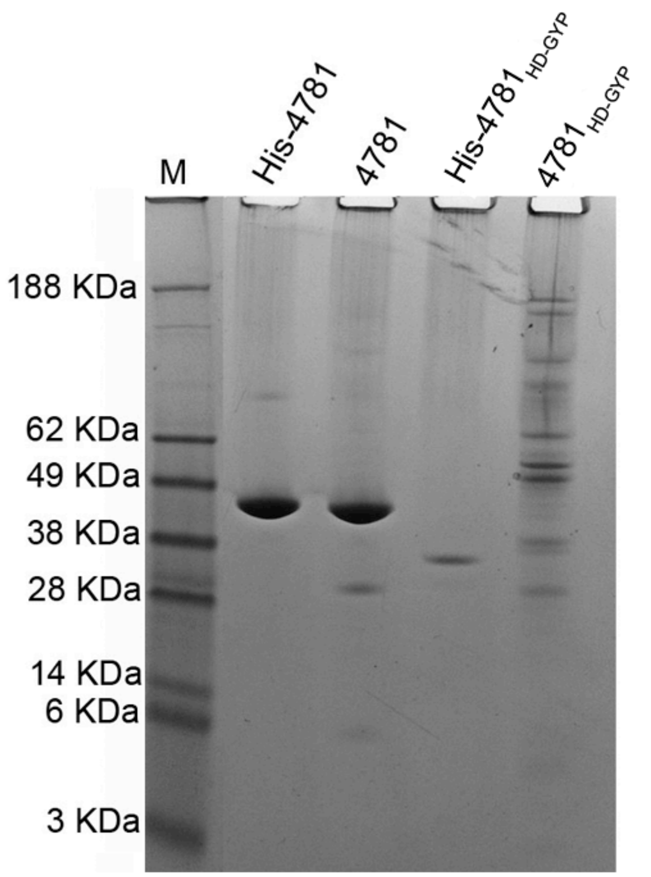


Figure S5. SDS-PAGE after proteolysis. Over-night incubation of His-tagged PA4781 with thrombin results in cleavage of the His-tag (lane 3) while the incubation of His-tagged $PA4781_{HD-GYP}$ (lane 4) results in protein degradation and dramatic aggregation (lane 5). His-tagged PA4781 was used as reference (lane 2). Each protein sample was incubated in 200 µl with 3 Units of thrombin (Sigma) overnight at room temperature. Protein concentration: 88 µM His-PA4781; 17 µM His-$PA4781_{HD-GYP}$.
